# Applying thermal therapy: Comparison of different commercially available heating devices to increase muscle temperature

**DOI:** 10.1113/EP092921

**Published:** 2026-01-18

**Authors:** Nada Nasir, Nathan Townsend, Marco Cardinale, Mariem Labidi, Sebastien Racinais

**Affiliations:** ^1^ Research and Scientific Support Department Aspetar Orthopedic and Sports Medicine Hospital Doha Qatar; ^2^ College of Health and Life Sciences Hamad Bin Khalifa University Doha Qatar; ^3^ Division of Targeted Interventions University College London London UK; ^4^ Department of Sports Exercise and Rehabilitation Northumbria University Newcastle Upon Tyne UK; ^5^ CREPS Montpellier Font‐Romeu Environmental Stress Unit Montpellier France; ^6^ DMEM Univ Montpellier INRAE Montpellier France

**Keywords:** heat therapy, hot‐water immersion, muscle temperature, short‐wave diathermy, water‐perfused suit

## Abstract

Although various medical devices are available for the purpose of heat therapy, their effect on muscle temperature remains unclear. This study compared the effects of a water‐perfused suit (WPS), short‐wave diathermy (SWD) and hot‐water immersion (HWI) on muscle, core and skin temperatures, along with perceived thermal discomfort. Ten healthy volunteers (four males and six females) were exposed to WPS, SWD or HWI for 1 h on three occasions, separated by 3–7 days, in a counterbalanced order. Muscle temperature increased with all devices (*P* < 0.0001, partial η^2^ = 0.55) but was lower after WPS in comparison to both SWD (*P* = 0.00656) and HWI (*P* = 0.00949). Core temperature was higher with HWI than with WPS (*P* ≤ 0.0104) and SWD (*P* ≤ 0.0213) from 20 min onwards. Although the average skin temperature over the thigh was lower with SWD than with HWI (*P* = 0.007, −1.2 [−2.0; −0.4]°C), the maximal local skin temperature was higher with SWD than with HWI (*P* = 0.0153, −0.7 [−1.4; −0.2]°C). Thermal discomfort was higher during HWI than during WPS (*P* ≤ 0.0159) and SWD (*P* ≤ 0.0130). In conclusion, only SWD and HWI increased muscle temperatures. SWD was able to increase local muscle temperatures comfortably, but the effects were localized. HWI can increase both peripheral and central temperatures easily, but the associated increases in core temperature might lead to hyperthermia‐induced discomfort.

## Abstract (highlights)

- **What is the central question of this study?**
  Do some commercially available heating devices increase intramuscular temperature more than others during 60 min of heating?
- **What is the main finding and its importance?**
  Muscle temperature increases to a greater extent with slow‐wave diathermy and hot‐water immersion than via a water‐perfused suit. However, hot‐water immersion leads to greater whole‐body thermal discomfort than slow‐wave diathermy. These results provide useful information that can be applied in a clinical rehabilitation setting.

## 1 INTRODUCTION

Although muscle function is known to be affected by acute muscle temperature changes (Racinais & Oksa, 2010), evidence has also emerged during the last two decades that repeated muscle heating could trigger chronic adaptations in skeletal muscle structure and function. Notably, in vitro (Goto et al., 2003) and animal (Uehara et al., 2004) studies demonstrated that repeated heat exposures can increase cell proliferation and muscle protein content. Moreover, animal studies showed that repeated heat exposures reduced muscle atrophy during immobilization (Naito et al., 2000) and promoted muscle regeneration post‐immobilization (Selsby & Dodd, 2005). More recently, repeated passive heat exposure was shown to limit muscle atrophy during immobilization in healthy humans (Hafen et al., 2019; Labidi et al., 2024). Therapeutic heating devices, such as a water‐perfused suit (WPS) and short‐wave diathermy (SWD), are thus gaining popularity in the field of musculoskeletal rehabilitation.

To optimize the physiological benefits induced by therapeutic heat stress, it has been suggested that muscle temperatures in the range 38.5°C–41°C should be attained (Yoshihara et al., 2013). For instance, in rodents subjected to varying degrees of environmental heat stress (37°C–41°C), anabolic‐related signalling was enhanced in a temperature‐dependent manner, with the most pronounced activation evident at 41°C (Yoshihara et al., 2013). In contrast, excessive hyperthermia can lead to adverse effects. In fact, continuous heat stress (>6 h at >41°C) was found to increase protein degradation and impair myoblast differentiation, resulting in poorly formed myotubes (Luo et al., 2000; Yamaguchi et al., 2010). In contrast, in healthy humans following 10 consecutive days of whole‐body passive heat stress, there was an increase in force‐generating capacity and contractile properties of lower‐limb muscles (Racinais et al., 2017), whereas a similar exposure protocol with only moderate localized heat stress did not confer any benefits, even after several weeks (Labidi et al., 2021). This is likely to be because moderate localized heat stress, such as that produced by a water‐perfused suit, might not activate hypertrophic signalling pathways within skeletal muscle as effectively as whole‐body hyperthermia (Ihsan et al., 2020). Interestingly, however, both local heating capable of increasing deep muscle temperature (Hafen et al., 2019) and whole‐body heating, which elevates both muscle and core temperature (Labidi et al., 2024), might induce therapeutic benefits, such as minimizing muscle atrophy during immobilization.

Despite the growing interest in passive muscle heating and the publication of several recent reviews discussing the benefits from a conceptual point of view (Ihsan et al., 2019; Kim et al., 2020; Lubrano et al., 2023; Rodrigues et al., 2020; Zanoli et al., 2024), there is no original investigation that directly compares increased muscle temperature triggered by various commercially available heating methods. Therefore, the aim of this study was to characterize the muscle temperature responses, especially the capacity to reach or exceed the 38.5°C threshold, along with other thermal responses, including core and skin temperatures, during 60 min of lower‐limb heating using three different commercially available techniques: (1) a water‐perfused suit (WPS); (2) short‐wave diathermy (SWD); and (3) hot‐water immersion (HWI). It was hypothesized that all three modalities would increase muscle temperature, but that SWD and HWI would induce a larger rise in intramuscular temperature than WPS, with HWI also eliciting greater core temperature elevation than other local devices.

## 2 MATERIALS AND METHODS

### 2.1 Participants

Ten healthy volunteers (four males and six females; age, 26.9 ± 5.1 years; body mass, 73.1 ± 19.8 kg; height, 174 ± 12 cm) participated in this study. This sample size was calculated with an α‐level of 0.05 and a power of 0.8, based on a previous study suggesting a difference of 0.8°C ± 0.8°C between WPS and a more aggressive passive heating (Ihsan et al., 2020). Inclusion criteria were healthy adults, body mass index of <30 kg/m^2^, not pregnant, not under medical treatment and not heat‐acclimated within the previous 3 months. Exclusion criteria were the presence of known contraindications to heat exposure and any metal implants (e.g., surgical screws). Participants were asked to avoid all exercise, caffeine and any medication that could affect body temperature, to maintain a similar food and fluid intake for ≥24 h preceding all tests and to avoid heavy meals 2 h preceding tests. This study complied with the principles of the *Declaration of Helsinki* and was approved by the Aspire Zone Foundation Institutional Review Board (approval #E2019000307). All participants signed the informed consent form after being briefed on the project details and associated risks.

### 2.2 Experimental design

All participants reported to the laboratory on three occasions, each separated by 3–7 days. Following the instrumentation (details below), participants underwent 60 min of one of the following three treatments, in a partly counterbalanced order.

#### 2.2.1 Water‐perfused suit

**FIGURE 1:**
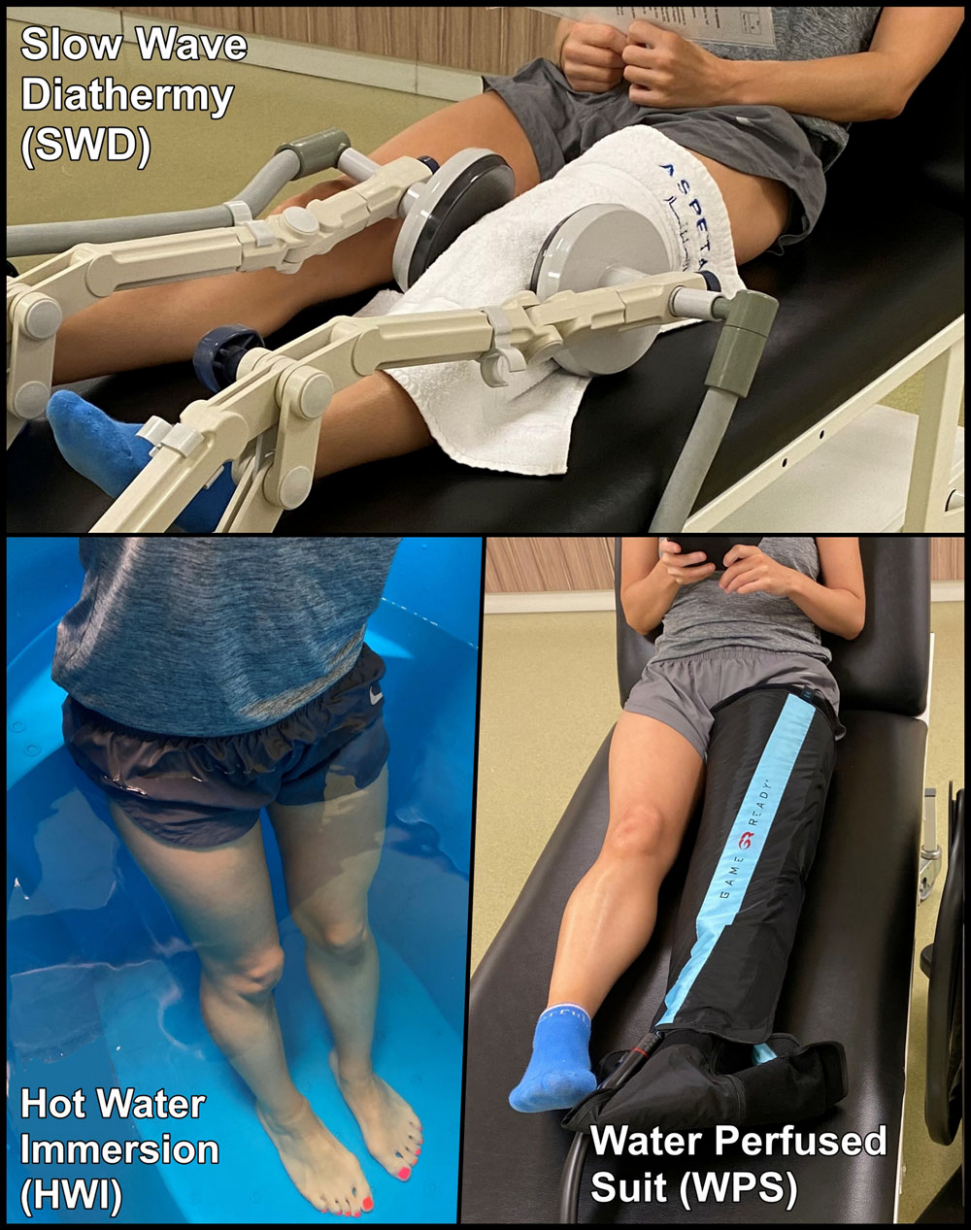
Devices for water‐perfused suit (WPS), short‐wave diathermy (SWD) and hot‐water immersion (HWI).

A water‐perfused suit (Game Ready Med4 Elite, CoolSystems Inc., USA) was wrapped around the left leg, covering from the foot to the hip, while the participant remained in a semi‐reclined position (Figure 1). The internal temperature of the suit was 42.3°C ± 1.4°C, verified using a calibrated digital thermometer inserted into the outer layer of the wrap to measure the temperature of the circulating water within the suit.

#### 2.2.2 Short‐wave diathermy

A short‐wave device (Enraf‐Nonius, Curapuls 970, the Netherlands) was applied on the left thigh with a frequency of 200 Hz and an intensity set at 10 (arbitrary units; as per the manufacturer's display). The frequency of 200 Hz corresponds to the manufacturer's recommended maximal pulse repetition frequency that ensures deep‐tissue heating while maintaining patient comfort. This frequency and intensity are consistent with prior clinical studies using comparable power densities (Hawkes et al., 2013).

The device was located on both sides of the thigh, in proximity to the skin but without contacting it (Figure 1). The participants remained in a semi‐reclined position. Participants were carefully instructed not to wear or have any metallic accessories/devices nearby.

#### 2.2.3 Hot‐water immersion

Participants were seated, immersed to the waist, in a water bath (iCool compact XP dual temperature, Australia; Figure 1). The water was continuously circulated and maintained at a constant temperature of 42.0°C ± 0.4°C. This temperature was chosen because it represents the upper limit of tolerability for prolonged (∼60 min) clinical immersion and is consistently reported to elevate muscle temperature to the therapeutic range of 38.5°C–40°C without inducing unsafe core hyperthermia (Brunt & Minson, 2021). All sessions were conducted at the same time of the day, in a room at ∼23°C and 57% relative humidity, and with the participants being instructed to consume 300–500 mL of water in the 2 h preceding the tests. Additionally, water was provided throughout the procedures, and participants were permitted to drink ad libitum.

### 2.3 Measurements

#### 2.3.1 Muscle temperature

Muscle temperature of the vastus lateralis was measured at rest and immediately after each intervention using an indwelling thermistor (MAC, Ellab, Hilleroed, Denmark), inserted under topical anaesthesia (EMLA cream, 5% lidocaine/prilocaine, Aspen, Ireland) at two‐thirds of the distance between the greater trochanter and patella border, at muscle depths of 3, 2 and 1 cm. The topical anaesthesia was applied during a 2 min break in the middle of the intervention to allow immediate measurement at the end of the intervention. The measurement site was identified and marked before starting the procedure. During the HWI, the topical anaesthesia was covered with a waterproof transparent polyethylene plaster to prevent water from removing the cream.

#### 2.3.2 Skin temperature

Skin temperature was measured in the middle of each intervention (i.e., before applying the topical anaesthesia for muscle temperature). A high‐resolution thermal camera (FLIR T600 480 × 360 pixel infrared resolution, thermal sensitivity < 40 mK, FLIR, Wilsonville, OR, USA) was used to measure the average skin temperature over the entire quadriceps, in addition to the maximal skin temperature reached over any part of the quadriceps. Thermal image acquisition was obtained with the participants standing still, 3 m in front of the camera, with all recommended methodological considerations remaining constant (i.e., participant demographics: age, sex and health status; environmental and camera conditions: camera calibration and specifications, camera distance and angle, ambient temperature, humidity, air movement, lighting, surface reflectivity, time of day; and recording and analysis process: region of interest selection, analysis software, analysis method and interpretation criteria) (Moreira et al., 2017). The camera and software analysis were set for human skin emissivity (ε = 0.98) to analyse the temperature of the quadriceps, with corrections for distance and reflected temperature of the environment applied. Skin was towel‐dried before measurements. The thermal images were analysed using the Thermal Studio PRO software (FLIR Systems, OR, USA).

#### 2.3.3 Core temperature

Core temperature was measured every 5 min via an ingestible capsule (e‐Celcius, BodyCap, Caen, France), which was wirelessly connected to a recording device with a sampling period of 30 s (e‐Viewer, BodyCap, Caen, France). The core temperature pill was ingested 30 min before the interventions. Participants were allowed to consume room temperature water ad libitum, but only immediately following a measurement reading, which therefore allowed a 5 min period for temperature equilibrium prior to the next. Furthermore, all data were visually inspected, and artefacts were removed following established recommendations (Byrne & Lim, 2007).

#### 2.3.4 Perceptual responses

Self‐perceived thermal discomfort was assessed every 10 min throughout each intervention using a four‐point validated scale (from 0 = comfortable to +3 = very uncomfortable) (Zhang et al., 2004).

### 2.4 Statistical analysis

Data were coded in SPSS v.21.0 (SPSS, Chicago, IL, USA). The effects of device (WPS, SWD and HWI) and depth (3, 2 and 1 cm) were analysed for muscle temperature using a two‐way ANOVA for repeated measures. The assumption of sphericity was verified using Mauchly's *W* preceding all statistical analysis, and a Greenhouse–Geisser correction was applied where appropriate. In the event of a significant interaction (condition × time), Tukey's adjustment was used to compare the effect of condition at each time interval and the effect of time within each condition. The effect of time on skin temperature was analysed using a one‐way ANOVA for repeated measures. The effect of device and time on thermal discomfort was analysed using a non‐parametric one‐way ANOVA for repeated measures (Friedman's test), and the Durbin–Conover *post hoc* test was used to examine pairwise comparisons. Effect sizes for the two‐way ANOVA main effects are described in terms of partial η^2^ (η^2^ ≥ 0.06 representing a moderate effect and η^2^ ≥ 0.14 a large effect). Mean differences for pairwise comparisons are reported with 95% confidence intervals. Data are reported as the mean (SD), and the level of statistical significance was set at *P* < 0.05.

## 3 RESULTS

### 3.1 Muscle temperature

**FIGURE 2:**
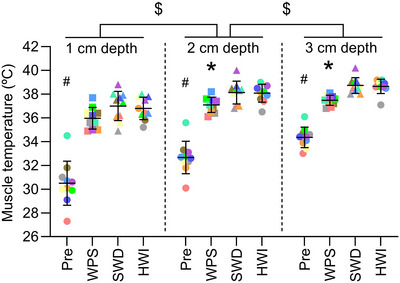
Muscle temperature was measured at three different depths after 60 min of treatment with a water‐perfused suit (WPS), short‐wave diathermy (SWD) or hot‐water immersion (HWI). ^#^Pre measure is different to post intervention for each experimental condition; ^$^Deeper temperatures greater than more superficial; *WPS was lower than both SWD and HWI, *P *< 0.05, *n* = 10.

All devices led to an increase in muscle temperature in comparison to resting muscle temperatures (WPS +4.3 [2.9; 5.7]°C, HWI +5.3 [3.9; 6.7]°C and SWD +5.5 [4.1; 6.8]°C, all *P* < 0.0001). Thus, there was a significant main effect of device on muscle temperature (*P* < 0.000803, η^2^ = 0.55). *Post hoc* analysis revealed a significantly lower muscle temperature after WPS than after both SWD (*P* = 0.00656, −1.1 [−1.9; −0.3]°C) and HWI (*P* = 0.00949, −1.0 [−1.7; −0.2]°C; Figure 2). There was also a main effect of depth on muscle temperature (*P* < 0.0001, η^2^ = 0.93), with higher values at a depth of 3 cm than at 2 cm (*P* = 0.00294, +0.5 [0.2; 0.8]°C), and at 2 cm compared with 1 cm (*P* < 0.0001, +1.2 [1.0; 1.4]°C; Figure 2). There was no significant interaction between the effect of devices and depth of measurement of muscle temperature (*P* = 0.642, η^2^ = 0.05).

### 3.2 Skin temperature

**FIGURE 3:**
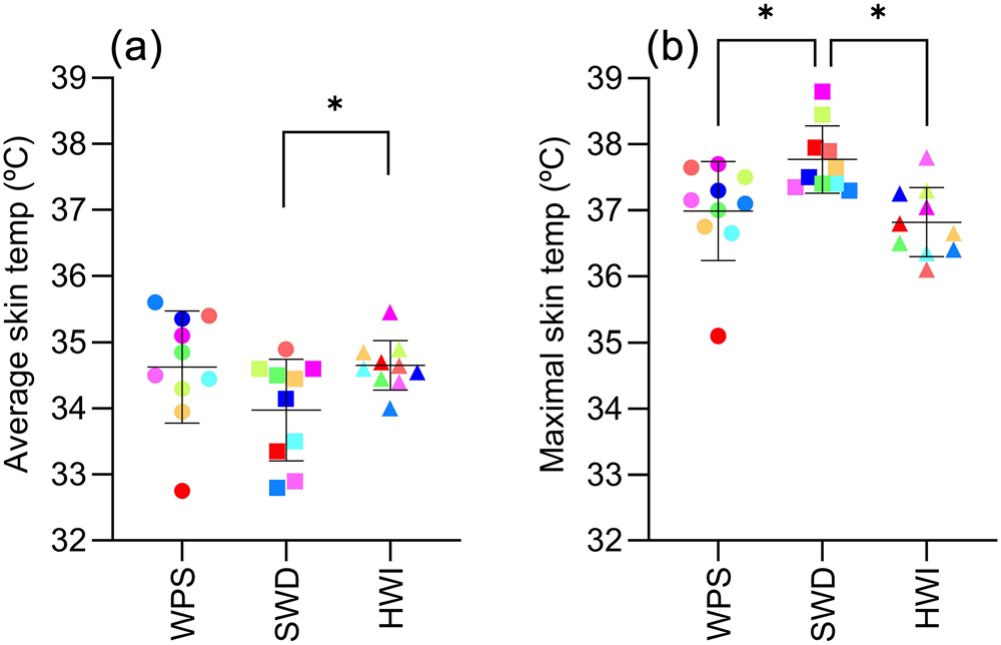
Average (a) and maximal (b) skin temperatures measured via thermal imaging of the thigh during treatment with a water‐perfused suit (WPS), short‐wave diathermy (SWD) or hot‐water immersion (HWI). Note that ‘average skin temperature’ represents the mean temperature across the entire quadriceps region of interest, whereas ‘maximal skin temperature’ refers to the highest pixel temperature recorded within the region. *P < 0.05, *n* = 10.

The average thigh skin temperature depended on the device (*P* = 0.0485, η^2^ = 0.29; Figure 3), with a lower average value with SWD in comparison to HWI (*P* = 0.0153, −0.7 [−1.4; −0.2]°C). The thigh maximal skin temperature also depended on the device (*P* = 0.00265, η^2^ = 0.48), but with a higher maximal value with SWD in comparison to HWI (*P* = 0.00351, +0.95 [0.5; 1.4]°C) and WPS (*P* = 0.0331, +0.78 [0.2; 1.3]°C).

### 3.3 Core temperature

**FIGURE 4:**
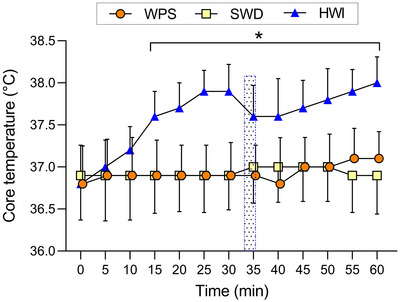
Core temperature during treatment with a water‐perfused suit (WPS), short‐wave diathermy (SWD) or hot‐water immersion (HWI). The dotted area represents the intermediate break for methodological purposes (see text for details). *HWI higher than both WPS and SWD, *P *< 0.05, *n* = 10.

There was a main effect of device (*P* < 0.0001, η^2^ = 0.71) and time (*P* < 0.0001, η^2^ = 0.64) on core temperature, with an interaction between device and time (*P* < 0.0001, η^2^ = 0.53). *Post hoc* comparisons showed that core temperatures were higher during HWI than during both WPS (*P* ≤ 0.0104) and SWD (*P* ≤ 0.0213; Figure 4) at all time points from 20 min onwards.

### 3.4 Perceptual responses

**FIGURE 5:**
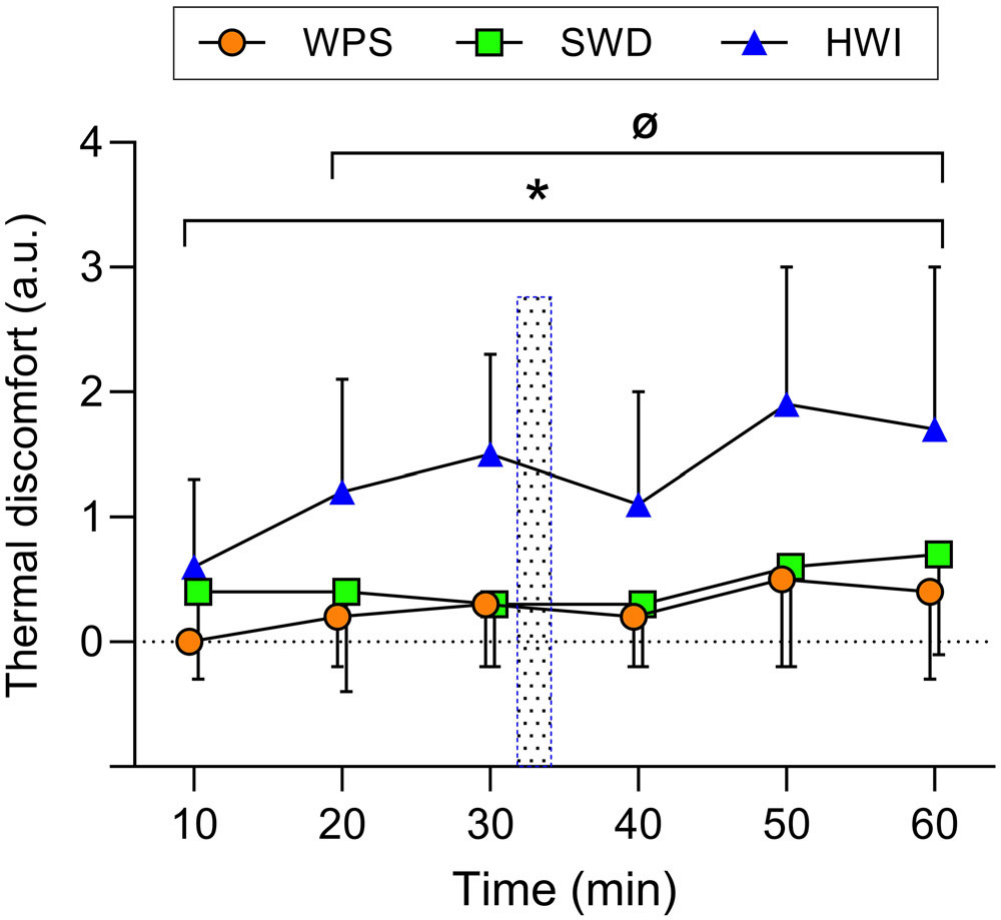
Thermal discomfort during treatment with a water‐perfused suit (WPS), short‐wave diathermy (SWD) or hot‐water immersion (HWI). The dotted area represents the intermediate break for methodological purposes (see text for details). *HWI had higher thermal discomfort than WPS at the time points indicated (*P *< 0.05). ^ø^HWI had higher thermal discomfort than SWD (*P *< 0.05), *n* = 10.

Thermal discomfort increased over time only for HWI and WPS, whilst a significant increase occurred sooner for HWI (30 vs. 10 min; *P* = 0.00525) than for WPS (50 min vs. 10 min; *P* = 0.0179; Figure 5). Furthermore, thermal discomfort was higher in HWI compared with WPS at all time points (all *P* ≤0.0159) and higher than SWD at all time points except 10 min (all *P* ≤0.0130).

## 4 DISCUSSION

The purpose of this study was to compare the thermal responses to three heating modalities commonly used to apply heat therapy. Our main results showed that: (1) SWD and HWI were both more effective at increasing muscle temperature than WPS; (2) SWD induced a higher maximal local skin temperature but an overall lower average skin temperature over the thigh than HWI; (3) HWI increased core temperature more than SWD and WPS; and (4) thermal discomfort was higher with HWI than with both SWD and WPS.

### 4.1 Water‐perfused suit

WPS are commonly available in athletic and rehabilitation settings. Although most WPS systems are designed to provide cold exposure, often combined with compression, newer systems also allow the provision of heat (Figure 1). The main benefits of WPS systems are practicability, relative safety and the absence of strong contraindications. They also provide consistent heat delivery without overwhelming systemic thermoregulatory capacity (González‐Alonso et al., 1999). Indeed, the WPS conditions were considered comfortable by the participants (Figure 5), with thermal discomfort scores near ‘comfortable’ (0.3 ± 0.5). The comfort associated with this device can probably be explained by the absence of marked increases in core temperature (Figure 4). Indeed, core temperature showed only minor increases after 55 min, consistent with previous studies that reported increases during protocols lasting 2 h (Ko et al., 2020), but not for 1 h (Ihsan et al., 2020). Although this relatively stable core temperature benefits comfort, it might limit the physiological effects associated with heat exposure, in terms of both increasing muscle temperature (see below) and the potential systemic cardiovascular benefits of heat stress for immobilized athletes (Ihsan et al., 2019). Of note, a study that involved a single‐leg WPS protocol similar to the present investigation did not report increased core temperature (Heinonen et al., 2011; Ihsan et al., 2020), whereas applying the WPS over a larger body surface area could overcome this limitation (Gamio, 2011; Gates, 2015).

The present data showed that WPS triggered an increase in muscle temperature (+4.3 [2.9; 5.7]°C) without a concomitant increase in core temperature, confirming previous data (Kellogg et al., 1998). However, the values reached were lower than the temperatures obtained with other devices and remained <38°C (Figure 2). Such muscle temperatures are unlikely to induce significant muscle remodelling, as previously suggested (Ihsan et al., 2020). In summary, WPS is a tool that is easy to use but appears to have limited effect on deep muscle temperature.

### 4.2 Slow‐wave diathermy

Although SWD is not as widely used as WPS, both techniques are commercially available and already present in physiotherapy centres (Figure 1). Similar to WPS, the present data showed that SWD had limited effect on core temperature (Figure 4) and thermal discomfort (Figure 5). SWD requires only a power supply for heat to be generated via a magnetic field, whereas WPS also requires a water supply. However, SWD magnetic fields require caution in certain situations, such as in the presence of pregnancy, metal implants, pacemakers, teleph70174ones and other artefacts likely to be influenced by a magnetic field and place the patient at risk of injury (Draper, 2006; Ouellet‐Hellstrom & Stewart, 1993).

Importantly, SWD induced larger increases in muscle temperature in comparsion to WPS (Figure 2), with average temperature values of 38.1°C at 2 cm depth and 38.7°C at 3 cm depth. Thus, SWD appears to be a suitable device when an increase in muscle temperature is targeted, confirming previous studies reporting benefits from deep muscle heating during therapeutic interventions such as stretching and joint mobilization (Hawkes et al., 2013; Rodrigues et al., 2020). Moreover, SWD has shown positive heat therapy responses in immobilized patients, including the preservation of skeletal muscle mitochondrial function and reduced muscle atrophy (Hafen et al., 2019). Of note, the peak increase in skin temperature was greater locally, but average skin temperature was lower than with other devices (Figure 3). These findings align with previous reports suggesting that SWD could heat deeper tissues with minimal skin overheating in comparison to superficial heating methods and suggest that the increase in muscle temperature was likely to be limited to the area directly under the coils. Of note, although the exact depth might vary depending on the subcutaneous layer (Rodrigues et al., 2024), the needle placement and depth were consistent between trials (i.e., repeated measures), and the differences between depths remained 1 cm within trial (i.e., 1 cm marks on the needle).

### 4.3 Hot‐water immersion

HWI is a cost‐effective intervention that can be implemented using either a normal bathtub or a dedicated clinical water‐immersion tub with temperature control (Figure 1). The present data confirmed that HWI increases core temperature (Hu et al., 2012; Rodrigues et al., 2020; Thomas et al., 2017) and showed that this increase was larger than with the other tested devices (Figure 4). Thus, aligned with existing literature (Morrison et al., 2004), HWI exaggerated thermal discomfort of the participants (Figure 5). Although the scores were statistically higher with HWI than with both WPS and SWD, peak values did not reach the scale maximum (on average across all participants), and clinical practice indicates that patients tolerate 60 min of HWI better than 60 min of a hot room at 48°C–50°C. Furthermore, HWI induced a higher average skin temperature than SWD over the quadriceps (Figure 3), suggesting that its effects could benefit a larger muscle area. Indeed, the greater increase in core temperature and thermal discomfort observed with HWI might be explained, in part, by the larger surface area exposed to heat, in comparison to WPS and SWD. As shown in Figure 1, the HWI conditions involved submersion to the waist, whereas the other methods targeted only the lower‐limb or thigh region. Although this design cannot control precisely for differences in thermal energy transfer between the three heating techniques, it does reflect an ecologically relevant use of these methods. Hence, the present results reflect outcomes pertaining to the practical application of each device in a clinical setting. Future studies might consider normalizing the heated surface area to isolate the intrinsic effects of each modality.

Importantly, considering that heat transfers 24 times faster in water than in air, HWI is capable of increasing muscle temperature in 15 min only (Brunt & Minson, 2021; Faulkner et al., 2017; Hawkes et al., 2013; Petrofsky et al., 2009; Rodrigues et al., 2020). The present data showed that HWI produced a similar increase in muscle temperature to SWD, with average values of 38°C at 1 and 2 cm depth and 38.7°C at 3 cm depth (Figure 2). Therefore, the clinical effects of both methods are likely to be similar, at least for skeletal muscle adaptations. Moreover, it has been shown that HWI enhances circulation and metabolic responses owing to the rapid transfer of heat through the skin (Versey et al., 2013; Wilcock et al., 2006). In addition, combining an increase in core temperature with an increase in muscle temperature could also stimulate muscle adaptations (Ihsan et al., 2020) and facilitate systemic adaptations (Ihsan et al., 2019). Additionally, recent work suggests a potential therapeutic benefit in immobilized patients using whole‐body heat exposure by minimizing muscle atrophy and loss in muscle force‐generating capacity (Labidi et al., 2024).

### 4.4 Limitations

Any intervention effects depend upon the characteristics (i.e., intensity and duration) of the exposure protocol; therefore, a limitation of the present experimental design relates to the variety and scope of the protocols chosen. Given that the heating devices examined in this study rely on different modes of energy transfer (conduction vs. electromagnetic) and application surface area, perfect stimulus standardization was not feasible. This represents an unavoidable limitation in the present experimental design; hence, the between‐device comparisons reflect the physiological responses to clinically relevant exposure protocols (including adherence to manufacturers’ safety guidelines) rather than thermally matched stimuli. This point was recently addressed in a review, which emphasized the need to report tissue temperatures rather than solely device settings when comparing heat therapy modalities (Rodrigues et al., 2024). Although it is acknowledged the choice of exposure protocol could impact the results, for SWD we opted for the maximum thermal setting of the device (200 Hz; intensity, 10 a.u.), and for HWI the temperature (42°C) was set to the upper limit of participant tolerability established from prior research (Dablainville et al., 2025; Ihsan et al., 2020; Labidi et al., 2024). The WPS temperature was therefore also set to 42°C to match the HWI conditions. Moreover, all exposure durations were matched to 60 min, again based on prior research (Ihsan et al., 2020; Labidi et al., 2024) and manufacturers’ use guidelines. In each case, the exposure protocol therefore represents the upper limit of acceptable use in a practical or clinical scenario. Future studies could examine the effect of different thermal stimuli over shorter/longer durations.

Another possible methodological limitation that could have impacted our results is the 30 min pre‐exposure duration after the core temperature pills were ingested, because in one study it was reported that this duration might be insufficient for complete gastric transit, which increases the likelihood that water ingestion could impact the temperature readings (Domitrovich et al., 2010). However, the total ad libitum fluid intake was relatively low in the present study (WPS, 66 ± 139 mL; SWD, 33 ± 69.6 mL; HWI, 355 ± 291 mL), and furthermore, participants typically only began water consumption after 30 min of exposure time, hence 60 min since ingestion of the core temperature pill. All data were inspected for water ingestion artefact and removed where appropriate according to guidelines set out by Byrne and Lim (2007). It is also possible that baseline differences in hydration status could have impacted the physiological response to thermal stress. We attempted to control for this variable by using standardized pretesting fluid and food intake instructions to participants. Moreover, although the effect of hydration status on physiological responses to exercise‐induced thermal stress is well known (Périard et al., 2021), there is a lack of data during resting conditions. Any differences in our participant cohort are unlikely to have occurred systematically and therefore would have minimal impact on the study outcomes.

Lastly, although the study included both male and female participants, no specific controls were applied regarding the menstrual cycle phase for female participants. It is plausible that hormonal fluctuations across the cycle might influence thermoregulatory responses; for example, it is known that core body temperature tends to be higher in the luteal phase (Baker et al., 2020). However, it is unlikely that this would have altered the primary outcome variable (muscle temperature), because it appears that the primary mechanism of thermal energy transfer in the SWD and WPS conditions was locally applied conduction as opposed to a heat‐exchange mechanism via increased blood temperature, because the latter should also have induced a change in core temperature, but this did not occur. Although the present project aimed to provide an overview of thermal responses to lower‐limb heat application in a real‐world setting, examination of sex differences and/or the impact of the menstrual cycle on the physiological response to heat therapy in resting conditions could be a relevant avenue of future research.

### 4.5 Practical applications

In scenarios where a moderate rise in muscle temperature is sufficient or desired, WPS can effectively target superficial muscle temperatures without imposing perceptual discomfort. However, in most cases, therapeutic benefits depend on greater rises in muscle temperature (Ichinoseki‐Sekine et al., 2007), and therefore both SWD and HWI appear superior to WPS in that regard. If a local change in muscle temperature is sufficient and maintaining patient comfort is a priority, SWD provides an effective option by raising temperature locally without increasing core temperature. Alternatively, if a systemic response is targeted with increases in both muscle and core temperatures, then HWI represents the most effective intervention.

## 5 CONCLUSION

Water‐perfused suits, short‐wave diathermy and hot‐water immersion are commonly used techniques in physiotherapy. The present findings revealed differences in local and systemic thermal responses between methods, which indicates that they are not interchangeable. Although WPS was effective at comfortably increasing superficial muscle temperature, there was less impact on deep tissue temperature, which could limit the benefits for whole‐muscle tissue regeneration. In contrast, both SWD and HWI increased muscle temperature at all depths. Only HWI increased core temperature, which might facilitate a wider range of physiological adaptations associated with heat acclimatization; however, this was associated with greater thermal perceptual responses, thus potentially limiting patient acceptability. The specific differences in physiological and thermal responses between each modality should be considered when prescribing heat therapy in a clinical setting.

## AUTHOR CONTRIBUTIONS

Study concept and design: Nada Nasir, Mariem Labidi and Sebastien Racinais. Analysis and interpretation of data: Nada Nasir, Nathan Townsend and Sebastien Racinais. Drafting of the work and critical revision of the manuscript for important intellectual content: Nada Nasir, Nathan Townsend, Marco Cardinale, Mariem Labidi and Sebastien Racinais. All authors approved the final version of the manuscript and agree to be accountable for all aspects of the work in ensuring that questions related to the accuracy or integrity of any part of the work are appropriately investigated and resolved. All persons designated as authors qualify for authorship, and all those who qualify for authorship are listed.

## CONFLICT OF INTEREST

None declared.

## FUNDING INFORMATION

None.

## Data Availability

All individual data points for muscle and skin temperature, which were the primary outcomes, are presented within the manuscript figures. Additional de‐identified data may be obtained from the corresponding author upon reasonable request for legitimate research purposes, provided these requests comply with participant consent and institutional ethics requirements.
